# Supplementary material for: COVID-19 vaccine hesitancy among adults in Liberia, April–May 2021
Source: PLoS One. 2024 Apr 17;19(4):e0297089. doi: 10.1371/journal.pone.0297089 (PMC11023583; doi:10.1371/journal.pone.0297089)
Supplement: S1 File — (PDF) [file pone.0297089.s001.pdf]

COVID-19 Vaccine Hesitancy in Liberia

Informed Consent

This form should be signed by anyone who consents to participate in this study

Hello. My name is_______________ and I am __________________. We are conducting a study on the COVID-19 Vaccine Hesitancy in Liberia in collaboration with the Liberia Ministry of Health, National Public Health Institute and health partners.

As part of the study, we are asking healthcare workers and community members about COVID-19. The study focuses on both community members and health workers who lives and work in rural and urban communities in Montserrado and Nimba Counties.

If you agree to participate, we will ask you some questions. Your responses are important and your frank answers to these questions will help us design strategies to help improve the response to COVID-19 in Liberia. We will collect data using both electronic and paper-based data collection tool. The interview will take about 30 minutes.

Risks and Benefits
This study involves no more than minimal risk. You are free not to answer any questions for any reason and at any point in time you can discontinue. The benefit of taking part is that the information you provide will inform us on how to reduce the risk of COVID-19 infection in Liberia.

Confidentiality
We will delink personal identifiers from the report and only the study team will have access to the study database.

There is no compensation for taking part in this study.

At this time, if there are any additional questions/information you need to know about this study kindly ask me.

Attestation
I, the undersigned, understand the nature of the study, benefits, my right to voluntary participation, confidentiality, and withdrawal from the study without any victimization. I have had the opportunity to ask questions and answered to my satisfaction.

I hereby freely consent to take part in this study.

____
Initials of participant

____
Signature/Date

Thumbprint (If participant cannot sign)

Instruction: To the Interviewer Instruction:

Introduce yourself to the participants, take consent and start the interview.

---

i. **Name of Data Collector** ____________________

ii. Have you obtained informed consent?

- ◯ Yes
- ◯ No

iii. Is interviewee wearing a mask?

- ◯ Yes
- ◯ No (If No, Skip Qiii)

iii. Is interviewee mask covering the nose and mouth?

- ◯ Yes
- ◯ No

iv. Interview Start Time

v. Date of interview

vi. Geo-coordinates

vii. Type of Respondent

- ◯ On Street
- ◯ Household
- ◯ Health facility

Section A: IDENTIFYING/DEMOGRAPHIC CHARACTERISTICS

1. County

- ◯ Montserrado
- ◯ Nimba
- ◯ Other

1 (i). Health District

2. Name of Community

3. Are you a member of the community?

- Yes
- No

4. Are you a Health Worker?

- Yes
- No

5. Health facility name

6. Health facility type

- GOL
- Non-GOL
- NGO

7. Age (as at last birthday)

8. Sex

- Male
- Female

9. Religion

Christianity Islam Traditional religion

Atheist None Other

Other (specify)

10. Highest level of education completed

a. No formal education b. Primary school c. Junior High School

d. Senior High School e. Diploma/Associates/Technician f. Bachelor's degree

g. Master's degree h. PHD i. Other

Other (specify)

---

11. Marital status

a. Single b. Married c. Divorce

d. Widow/Widower e. Separated f. Co-habiting g. Other

Other (specify)

---

12. Employment status

---

a. Employed b. Self- employed c. Unemployed
d. Learning a trade e. Student f. Other

Other (specify)

---

13. If employed or self-employed, Occupation

Section B: COVID-19 PREVENTION

15. Is there anything you are doing to prevent COVID-19 infection?

a. Yes b. No c. I don't know d. Refused to answer

15b. No (If No, why not? _ and skip to Q17)

---

16. If yes, which preventive measure do you practice? (Tick all that apply)

a. By receiving Coronavirus vaccine b. Regular hand washing with soap
c. Frequently using water or alcohol rub d. Avoid touching of eyes, nose, and mouth
e. Putting on face and nose mask f. Avoid being in a crowded public place
g. Maintaining social distance h. Avoid handshake i. Don't Know j. Others

Others (specify)

---

17. How well do you feel that you are practicing the COVID-19 preventive measure of regular handwashing or sanitizingyour hands?

a. All the time b. Sometimes
c. Never

18. How well do you feel that you are practicing the COVID-19 preventive measure of social distancing?

To practice social or physical distancing, stay at least 6 feet (about 2 arm lengths OR 3 meters) from other people who are not from your household in both indoor and outdoor spaces.

a. All the time b. Sometimes
c. Never

19. How well do you feel that you are practicing the COVID-19 preventive measure of wearing of nose masks? a. All the time

b. Sometimes

c. Never

20. How strongly do you feel protected from getting infected with COVID-19 based on your actions?

a. Very well
b. Somewhat well
c. Neutral
d. Not well
e. Not at all

21. Have you ever been tested for Coronavirus?

a. Yes
b. No (Skip to Q23)
c. Refused to respond

22. Have you ever tested positive for Coronavirus?

a. Yes
b. No
c. Don't Know
d. Refused to respond

23. Do you know someone personally who has had COVID-19 infection?

a. Yes
b. No (Skip to Q25)
c. Refused to respond

24. If yes, who?

a. Family member
b. Friend
c. Someone in my community
d. Someone in my church/mosquee.
Work colleaguef. Other

Other (specify)

____

25. Do you know someone personally who has had the COVID-19 vaccine?

Yes
No (If No, move to Q27)
I don't know

26. If Yes, who?

Family member
Friend
Someone in my community
Someone in my church/mosqueWork colleagueOther

Other (specify)

____

____

27. **In general, do you think the government and health authorities are acting in the best interest of Liberians indealing with the coronavirus outbreak?**

a. Yes b. No Partially or sometimes

c. I don't know d. Refused to respond

28. **Give a reason for your choice in Question 27, Why?**

Section C: COVID-19 VACCINATION INTENTION

29. **What do you know about the COVID-19 vaccine?**

30. **Do you think COVID-19 vaccines are effective/useful in preventing COVID-19 infections?**

a. Yes b. No c. Don't know

d. Refused to respond

31. **Have you heard that the COVID-19 vaccines are available in Liberia?**

a. Yes b. No (Skip to Q33) c. Don't know d. Refused to respond

32. **If yes, how did you hear about it?**

a. Word-of-mouth b. Facebook c. Twitter d. WhatsApp, LINE or other messaging apps e. SMS

f. Patients and caregivers g. Healthcare provider h. Media (TV, radio, newspaper)

i. Community gathering j. Internet website k. Other

Other (specify)

33. **Who can receive the COVID-19 vaccine available in Liberia? (Tick all that apply)**

a. Everyone b. Health workers

c. Old people/people aged >60 years d. People with Hypertension/Diabetes Mellitus

e. I don't know f. Other

Other (specify)

34. **Have you taken a shot of the COVID-19 vaccine in Liberia in your arm?**

Yes

No

34(b). Will you take the COVID-19 vaccine?

- a. I would definitely not take it
- b. I am not likely to take it
- c. I am undecided
- d. I probably would want to take ite. I would definitely take itf. Other

Other (specify)

____

35. What is your reason? Why?

____

Section D: ATTITUDE TOWARD COVID-19 VACCINATION

Hints: In this section, I will like to know your opinion on attitude towards COVID-19 vaccination.

____

36. If you get the COVID-19 vaccine, is it possible to get the Coronavirus sickness?

- Yes
- No
- I don't know
- Other

Other (specify)

____

37. If you get the COVID-19 vaccine, is it possible to get infected with coronavirus, but the sickness will be less severe?

- Yes
- No
- I don't know

38. Do you think that COVID-19 vaccine can cause COVID-19 infection?

- Yes
- No
- I don't know

39. Is it necessary to get the vaccine if people in your environment are vaccinated?

- Yes
- No
- I don't know

40. Do you expect side effects or allergic reactions after getting COVID-19 vaccination.

◯ Yes ◯ No
◯ I don't know

41. Are you against vaccination in general?

◯ Yes ◯ No
◯ I don't know

42.Are you against the COVID-19 vaccine in particular?

◯ Yes ◯ No
◯ I don't know

43. Do you think protection received from the vaccine is stronger than protection from surviving the infection?

◯ Yes ◯ No
◯ I don't know

Section E: EXPOSURE TO INFORMATION

44. What do you usually do when someone shares a COVID-19 related information with you? (Select all that apply)

☐ a. I ignore it, stay silent ☐ b. Verify it using scientific evidence
☐ c. Share accurate resources with them ☐ d. Consult a friend before responding
☐ e. I don't know what to do in these situations ☐ f. Other

Other (specify)

___

45. Which information source(s) do you trust to verify information about the COVID-19 vaccine? Tick all that apply

☐ a. International health agencies (e.g., WHO, UNICEF, CDC) ☐ b. Local and international NGOs
☐ c. Ministry of Health ☐ d. National Public Health Institute
☐ e. Health care organizations (e.g., hospitals, clinics, nursing homes)
☐ f. Medical societies or professional associations ☐ g. Other

Other (specify)

___

Hints: Here, you will be asked questions on how you trust COVID-19 information from different sources.

___

46. Do you trust the information you see or hear on COVID-19 vaccine through the followingsources?

46(a). Word-of-mouth

- ○ Yes
- ○ No
- ○ I don't know
- ○ I don't have access

46(b). Facebook

- ○ Yes
- ○ No
- ○ I don't know
- ○ I don't have access

46(c). Twitter

- ○ Yes
- ○ No
- ○ I don't know
- ○ I don't have access

46(d). WhatsApp or other messaging apps

- ○ Yes
- ○ No
- ○ I don't know
- ○ I don't have access

46(e). SMS

- ○ Yes
- ○ No
- ○ I don't know
- ○ I don't have access

46(f). Radio

- ○ Yes
- ○ No
- ○ I don't know
- ○ I don't have access

46(g). Tik Tok

- ○ Yes
- ○ No
- ○ I don't know
- ○ I don't have access

46(h). YouTube

- ○ Yes
- ○ No
- ○ I don't know
- ○ I don't have access

46(i). Healthcare workers

- ◯ Yes
- ◯ No
- ◯ I don't know
- ◯ I don't have access

46(j). Media (TV, radio, newspaper)

- ◯ Yes
- ◯ No
- ◯ I don't know
- ◯ I don't have access

46(k). Community gathering

- ◯ Yes
- ◯ No
- ◯ I don't know
- ◯ I don't have access

46(l). Internet website

- ◯ Yes
- ◯ No
- ◯ I don't know
- ◯ I don't have access

46(m). Online News websites

- ◯ Yes
- ◯ No
- ◯ I don't know
- ◯ I don't have access

46(n). National Government officials /leaders

- ◯ Yes
- ◯ No
- ◯ I don't know
- ◯ I don't have access

46(o). Local government officials/leaders

- ◯ Yes
- ◯ No
- ◯ I don't know
- ◯ I don't have access

46(p). Religious Leaders/community leaders

- ◯ Yes
- ◯ No
- ◯ I don't know
- ◯ I don't have access

46(q). Celebrities and influencers

- Yes
- No
- I don't know
- I don't have access

Section F: Instruction to Interviewer

47. Did the interviewee keep the nose and mouth mask on during the entire interview?

- Yes
- No

48. Any comments?

49. Interview End Time
