# Supplementary material for: COVID-19 vaccine hesitancy among adults in Liberia, April–May 2021
Source: PLoS One. 2024 Apr 17;19(4):e0297089. doi: 10.1371/journal.pone.0297089 (PMC11023583; doi:10.1371/journal.pone.0297089)
Supplement: Attachment — Submitted filename: Review comments.docx [file pone.0297089.s004.docx]

**Review comments**

The study provides valuable data that shows the level of COVID-19 vaccine hesitancy in Liberia. The manuscript is well written and I have no major issues with it. I have just one suggestion for the authors and I need some clarification on the ethical approval.

1. On page 3, first paragraph under background, the authors indicated that only three COVID-19 vaccines were available in Liberia. I suggest the authors indicate the names of those specific COVID-19 vaccines.
2. The authors indicated that the study was approved by the National Incident Management System for COVID-19 responses, Ministry of Health, Liberia. Is the National Incident Management System for COVID-19 response in Liberia an ethical review committee or board? If not, what was the approval for? Is the US CDC non-research determination clearance an ethical clearance? If not, what was the clearance for?
