# Supplementary material for: COVID-19 vaccine hesitancy among adults in Liberia, April–May 2021
Source: PLoS One. 2024 Apr 17;19(4):e0297089. doi: 10.1371/journal.pone.0297089 (PMC11023583; doi:10.1371/journal.pone.0297089)
Supplement: Attachment — Submitted filename: Vaccine_study.docx [file pone.0297089.s005.docx]

A very important paper that would contribute to understanding the attitude of individuals towards the COVID-19 vaccine uptake. The organization of the manuscript is very good. However, there are essential aspects of the paper that needs to be addressed.

Background:

The background was well written. Authors should change the > (i.e.>30%) and (<20%) symbol to words. Some of the statements require citation(s) as the information is not emanating from the authors.

**Methods:**

Study design and population.

What is rapid cross-sectional study - “rapid” should be deleted.

**Major revision**:

The sampling technique requires a major revision.

1. Was the study stratified by rural and urban. This should be clear in the write up.
2. Stage 2. A table indicating the population size of each community and the number sample from each community will aid easy understanding of what was actually done.
3. The sampling interval should be stated. If it varies by community due to the different population size of the communities, this should be indicated clearly.
4. Stage 3: looks confusing. What is the relevance of stage 3 to this current paper?
5. The section on street interviews can be deleted. It suggests the authors where selecting participants hazardly. It masks the scientific merit of the whole paper. Authors should look at it critically if it does not contribute to the paper, it has to go/deleted. Otherwise, it should be revised. Importantly, how many interviewers where stationed to conduct this street interviews. Are the authors suggesting that they are able to complete an interview before the tenth participant?

**Training of data collection**

One-day training is mostly considered inadequate. Did the authors pilot the tool with the research assistants?

**Data analysis.**

Authors did a multi-stage sampling and stratified, therefore an appropriate statistical analysis has to be performed. The study requires a complex data analysis.

**Results**

When starting a sentence with a figure, it should be in words. Authors should check all that and correct.

Table 2 can be deleted. All information can be found in table 3.

**Discussion**

The discussion needs major revision. The paper was poorly discussed. There was overelaboration of issues or study findings. The authors should consider the design of the study and its limitation. Moreover, authors make statements that was outside the scope of this paper and the study findings does not support. For example, “**The distribution of ownership of radio or television could be skewed to the middle- and high-income earners who could afford it**”. Did the authors check the distribution of radio or television in among their study population? There are number of such statements in the discussion. Authors should focus the discussion and write within the context of the study design and its findings.
