# Supplementary material for: COVID-19 vaccine hesitancy among adults in Liberia, April–May 2021
Source: PLoS One. 2024 Apr 17;19(4):e0297089. doi: 10.1371/journal.pone.0297089 (PMC11023583; doi:10.1371/journal.pone.0297089)
Supplement: Attachment — Submitted filename: Response to Reviewers.docx [file pone.0297089.s006.docx]

Liberia Field Epidemiology Training Program

African Field Epidemiology Network (AFENET), Monrovia, Liberia.
lblebo@afenet.net

December 2, 2022

Martin Nyaaba Adokiya , Ph.D.

Academic Editor,
PLOS ONE

**RE**: COVID-19 Vaccine Hesitancy among adults in Liberia, April-May 2021 [PONE- D-22-17733]

Dear Dr. Adokiya,

We are very grateful to you and the reviewers for the time and efforts you all put in to review our manuscript and for valuable suggestions to improve the understanding of the work.

We have revised the manuscript to address the points raised during the review process. Our point-by-point responses to all comments and suggestions are tabulated on the next page of this letter.

We hope you find our responses satisfactory and the manuscript now suitable for publication in PLOS ONE, even as we appreciate further suggestions where we failed to address observed concerns.

Sincerely,

Lily M. Sanvee-Blebo

Field Epidemiologist
Liberia Field Epidemiology Training Program, AFENET

[On behalf of all authors]

| **Section** | **Comments** | **Authors response** |
| --- | --- | --- |
| **Editor’s comment** |  |  |
|  | **1.** The author should clarify the authority of the ethical approval institution | Thank you for the thorough review of our manuscript and for useful suggestions to improve the understanding of the work. We have carefully gone through the comments and made modifications as suggested in the work. We have also responded to the comments here for ease of reference to the changes made in the work. We hope that the work is now acceptable for publication and will be available to respond to further clarification, if any. |
|  | **2.** The methods section should be improved including the study design, the sampling technique and the number of interviewers | The method section has been improved and appropriate information added |
|  | **3.** The discussion should be limited to scope and focus on the current study | Thank you, the discussion has been edited |
|  | **4.** The omitted references should be included in the revised manuscript | The omitted references have been added |
| **Journal Requirements** |  |  |
|  | **1.** Please ensure that your manuscript meets PLOS ONE's style requirements, including those for file naming. The PLOS ONE style templates can be found at  https://journals.plos.org/plosone/s/file?id=wjVg/PLOSOne_formatting_sample_main_body.pdf and  https://journals.plos.org/plosone/s/file?id=ba62/PLOSOne_formatting_sample_title_authors_affiliations.pdf | Thank you for the useful links. We have used the links to format our manuscript accordingly. |
|  | **2.** Thank you for stating the following in the Acknowledgments Section of your manuscript:  The study was funded by the US. Centers for Disease Control and Prevention (US. CDC) Liberia and World Health Organization (WHO) Liberia  We note that you have provided funding information that is not currently declared in your Funding Statement. However, funding information should not appear in the Acknowledgments section or other areas of your manuscript. We will only publish funding information present in the Funding Statement section of the online submission form.  Please remove any funding-related text from the manuscript and let us know how you would like to update your Funding Statement. Currently, your Funding Statement reads as follows:  The author(s) received no specific funding for this work.  Please include your amended statements within your cover letter; we will change the online submission form on your behalf. | The funding information has been removed from the manuscript and it is stated clearly in the revised cover letter. |
|  | **3.** We note that you have indicated that data from this study are available upon request. PLOS only allows data to be available upon request if there are legal or ethical restrictions on sharing data publicly. For more information on unacceptable data access restrictions, please see http://journals.plos.org/plosone/s/data-availability#loc-unacceptable-data-access-restrictions.  In your revised cover letter, please address the following prompts:  a) If there are ethical or legal restrictions on sharing a de-identified data set, please explain them in detail (e.g., data contain potentially sensitive information, data are owned by a third-party organization, etc.) and who has imposed them (e.g., an ethics committee). Please also provide contact information for a data access committee, ethics committee, or other institutional body to which data requests may be sent.  b) If there are no restrictions, please upload the minimal anonymized data set necessary to replicate your study findings as either Supporting Information files or to a stable, public repository and provide us with the relevant URLs, DOIs, or accession numbers. For a list of acceptable repositories, please see http://journals.plos.org/plosone/s/data-availability#loc-recommended-repositories. | We have updated this part of the manuscript. All data are available within the manuscript and its supporting information. |
|  | **4.** Your ethics statement should only appear in the Methods section of your manuscript. If your ethics statement is written in any section besides the Methods, please delete it from any other section. | Thank you for this observation. We have deleted the ethical statement in the Acknowledgement section. |
| **Reviewer 1 Comments** |  |  |
|  | **1.** On page 3, first paragraph under background, the authors indicated that only three COVID-19 vaccines were available in Liberia. I suggest the authors indicate the names of those specific COVID-19 vaccines. | The names of the vaccines have been added to the paragraph in track changes. (Page 3) |
|  | **2.** The authors indicated that the study was approved by the National Incident Management System for COVID-19 responses, Ministry of Health, Liberia. Is the National Incident Management System for COVID-19 response in Liberia an ethical review committee or board? If not, what was the approval for? Is the US CDC non-research determination clearance an ethical clearance? If not, what was the clearance for? | The Incident Management System of COVID- 19 comprises the leadership of the Ministry of Health (Minister and Deputy Ministers) and the National Public Health Institute of Liberia (Director General). It was the body charged with the responsibility to approve all COVID-19-related studies during the response. Other COVID-19-related studies undertaken and approved by this body during this period have also been published  Acquiring the US. CDC clearance is a process that needs to be completed based on the policy of the organization before the study was conducted. However, it is not  relevant to the writing of this paper and has been deleted. |
| **Reviewer 2 comments** |  |  |
|  | **1.** The background was well-written. Authors should change the > (i.e.>30%) and (<20%) symbol to words. Some of the statements require citation(s) as the information is not emanating from the authors. | The symbols have been changed to words and new citations have been added. |
|  | **2.** Study design and population. What is rapid cross-sectional study - “rapid” should be deleted | The word “rapid” has been deleted |
|  | **3.** Was the study stratified by rural and urban. This should be clear in the write up. | The study was conducted in two of the 15 counties of Liberia. In each county, the communities were stratified into urban and rural communities. The sentence has been rephrased to provide more clarity |
|  | **4.** Stage 2. A table indicating the population size of each community and the number sample from each community will aid easy understanding of what was actually done. | The authors appreciate the comments. The stage has been restructured to better communicate what was done. It now reads, “The first household was selected from the community landmark by simple random sampling within the sampling interval. The sampling interval for each community was obtained by dividing the number of households with the sample sizes allocated to the community segment of the study. The interval varied from one community to the other based on number of households in each community.” |
|  | **5.** The sampling interval should be stated. If it varies by community due to the different population size of the communities, this should be indicated clearly | As explained above, the sampling interval vary from community to community depending on their population (household) size. However equal number of respondents were sampled from each community. |
|  | **6.** Stage 3: looks confusing. What is the relevance of stage 3 to this current paper? | The authors appreciate the comment. The study unit of study was the individual not the household. So stage 3 explained how an individual is sampled within the household. It has been edited to better represent that. |
|  | **7.** The section on street interviews can be deleted. It suggests the authors where selecting participants hazardly. It masks the scientific merit of the whole paper. Authors should look at it critically if it does not contribute to the paper, it has to go/deleted. Otherwise, it should be revised. Importantly, how many interviewers where stationed to conduct this street interviews. Are the authors suggesting that they are able to complete an interview before the tenth participant? | This section of the work has been deleted as requested. |
|  | **8.** One-day training is mostly considered inadequate. Did the authors pilot the tool with the research assistants? | The data collectors that collectors were already part of the response team in the field, involved in other trainings conducted previously. The one-day training focused on the protocol and how to select and administer the study tool. There was a pretesting of the study instrument after the training prior to the data collection. |
|  | **9.** Authors did a multi-stage sampling and stratified, therefore an appropriate statistical analysis has to be performed. The study requires a complex data analysis | The authors agree with the reviewer’s comment on the need for complex data analysis however, our primary indent was not to estimate the prevalence of the hesitancy at subcounty and community level so we did not apply the take the route. The stratification was done to better represent the different segment of the society in the sample. |
|  | **10.** When starting a sentence with a figure, it should be in words. Authors should check all that and correct. | The corrections have been made in the manuscript. |
|  | **11.** Table 2 can be deleted. All information can be found in table 3. | The crude odds ratio from Table 2 has been merged to table 3 and the old table 2 deleted. |
|  | **12.** The discussion needs major revision. The paper was poorly discussed. There was overelaboration of issues or study findings. The authors should consider the design of the study and its limitation. Moreover, authors make statements that was outside the scope of this paper and the study findings does not support. For example, “**The distribution of ownership of radio or television could be skewed to the middle- and high-income earners who could afford it**”. Did the authors check the distribution of radio or television in among their study population? There are number of such statements in the discussion. Authors should focus the discussion and write within the context of the study design and its findings. | The section has been reviewed and the offending aspects removed. |
