# Supplementary material for: COVID-19 vaccine hesitancy among adults in Liberia, April–May 2021
Source: PLoS One. 2024 Apr 17;19(4):e0297089. doi: 10.1371/journal.pone.0297089 (PMC11023583; doi:10.1371/journal.pone.0297089)
Supplement: Attachment — Submitted filename: RE-Vaccine hesistancy.docx [file pone.0297089.s008.docx]

**RE: COVID-19 Vaccine Hesitancy among adults in Liberia, April – May 2021**

General comments: This is a well written manuscript and addressed an important issue regarding vaccination in Liberia. The authors have addressed most of the earlier raised issues satisfactorily. However, there are still a few areas that need some improvements.

**Abstract**

Line 37: “multiple logistic regression” kindly correct as binary logistic regression.

**Methods**

Lines 102-103: Montserrado is considered a primarily urban county “(61% 1967/3230 seven districts, 22 zones)” this is a bit unclear? What does 1967/3230 indicated? while Nimba County “is predominantly rural (11% (159/1471) six districts)” This is unclear? What does 159/1471 six districts?

Line 119: “The sample size was calculated using the Kish and Leslie formulae (ref).” Kindly insert ref

Line 121: “design effect of 2” what does design effect of 2 meant? Is this part of the formula?

Line 178: “multiple logistic regression” What the authors did was simple binary logistic regression and should be corrected as such.

**Results:**

Line 200: “SMS” write in full.

Line 210-212: “When considering the effect of other variables in the model, respondents aged more than 55 years (aOR:0.5; 95% CI: 0.2–0.9; p=0.043) were two times more likely to be COVID-19 vaccination hesitant compared to those aged 45–54 years” This statement is at variance and may confuse the reader. It is ether you adjust your reference in the Table for positive odds ratio as reported in the prose or you interpreted as stated in Table 2 in lesser effects.

Line 224: Table 2. “Multivariate association” This is multivariable and not multivariate

**Discussion**

Lines 271-273: “With regards to age, 15-24 year-olds were more likely to express hesitancy to take the COVID-19 vaccine because the disease is more lethal to persons older and thus they did not see themselves at risk of getting the disease.” Your findings do not support this assertion/discussion. Indeed, it is contrary as vaccine hesitancy was more in those aged more than 55 years! Check your Table 2 results.

Line 276: “statistically significant predictor of the hesitancy of the COVID-19 vaccine” kindly replace with association- a cross sectional data can not give you a predictor. You may wish to read more about predictive models.

Line 278: “Urban dwellers were usually more likely to have mobile phones and engage in social media and more likely to obtain information from other sources” This statement is hanging? Not link with the predecessor and how does it lead to VH?

Line 280: “Our study is not without limitations. Our sample size was small and included only two of Liberia’s fifteen counties. Nevertheless, these counties accounted for 83% of all reported COVID-19 cases in the country” These statements are not limitation. Your sample size of 800 plus is adequately powered and sampling two major districts comprising of urban and rural areas out of 15 should provided an adequate representation and can not be a study limitation. Your study limitations may be lack of in-depth analysis of reasons for VH, which will require a qualitative study approach and also lack of follow up to see how many people will eventually get vaccinated.

Line 282: “The survey provides information needed to strengthen and increase the impact of future planned mass vaccination campaigns” Move to the end of conclusion

Overall while I understood time has caught up with your data, kindly compared your key findings with other African studies e.g Babatunde Oluwatosin Ogunbosi et al. COVID-19 vaccine hesitancy in six geopolitical zones in Nigeria: a cross-sectional survey. Pan African Medical Journal. 2022;42(179). 10.11604/pamj.2022.42.179.34135
