# Supplementary material for: COVID-19 vaccine hesitancy among adults in Liberia, April–May 2021
Source: PLoS One. 2024 Apr 17;19(4):e0297089. doi: 10.1371/journal.pone.0297089 (PMC11023583; doi:10.1371/journal.pone.0297089)
Supplement: Attachment — Submitted filename: Response to Reviewers docx.docx [file pone.0297089.s009.docx]

Liberia Field Epidemiology Training Program
African Field Epidemiology Network (AFENET), Monrovia, Liberia.
lblebo@afenet.net

October 20, 2023

Omar Enzo Santangelo, Ph.D.,
Academic Editor,
PLOS ONE

**RE**: COVID-19 Vaccine Hesitancy among adults in Liberia, April-May 2021 [PONE- D-22-17733]

Dear Dr. Santagelo,

We are very grateful to you and the reviewers for the time and efforts you all put in to review our manuscript and for valuable suggestions to improve the understanding of the work.

We have revised the manuscript to address the points raised during the review process. Our point-by-point responses to all comments and suggestions are tabulated on the next page of this letter.

We hope you find our responses satisfactory and the manuscript now suitable for publication in PLOS ONE, even as we appreciate further suggestions where we failed to address observed concerns.

Sincerely,

Lily M. Sanvee-Blebo

Field Epidemiologist
Liberia Field Epidemiology Training Program, AFENET

[On behalf of all authors]

| Section | Comments | Authors response |
| --- | --- | --- |
| Reviewer # 3 |  |  |
| 1 | Abstract Line 37: “multiple logistic regression” kindly correct as binary logistic regression. | Thanks for your comment, the changes have been made accordingly |
| 2 | Methods Lines 102-103: Montserrado is considered a primarily urban county “(61% 1967/3230 seven districts, 22 zones)” this is a bit unclear? What does 1967/3230 indicated? while Nimba County “is predominantly rural (11% (159/1471) six districts)” This is unclear? What does 159/1471 six districts? | The sentence has been rephrased to provide more clarity. The percentage represents the communities |
| 3 | Line 119: “The sample size was calculated using the Kish and Leslie formulae (ref).” Kindly insert ref | This has been added. |
| 4 | Line 121: “design effect of 2” what does design effect of 2 meant? Is this part of the formula? | The design effect of 2 indicates that the survey design has introduced some degree of clustering or stratification that increases the variance of the estimates, which means that the estimates are less precise than they would be with a more efficient design. It is not inherently part of the formular since it is a function of the sampling strategy adopted. |
| 5 | 5. Line 178: “multiple logistic regression” What the authors did was simple binary logistic regression and should be corrected as such. | This has now been corrected. |
| 6 | Line 200: “SMS” write in full | The acronym has been written in full, thank for the comment |
| 7 | Line 210-212: “When considering the effect of other variables in the model, respondents aged more than 55 years (aOR:0.5; 95% CI: 0.2–0.9; p=0.043) were two times more likely to be COVID-19 vaccination hesitant compared to those aged 45–54 years” This statement is at variance and may confuse the reader. It is ether you adjust your reference in the Table for positive odds ratio as reported in the prose or you interpreted as stated in Table 2 in lesser effects. | The sentence has now been rephrased as in the table to limit the risk of misunderstanding. |
| 8 | Line 224: Table 2. “Multivariate association” This is multivariable and not multivariate | The correction has been effected. |
| 9 | Lines 271-273: “With regards to age, 15-24 year-olds were more likely to express hesitancy to take the COVID-19 vaccine because the disease is more lethal to persons older and thus they did not see themselves at risk of getting the disease.” Your findings do not support this assertion/discussion. Indeed, it is contrary as vaccine hesitancy was more in those aged more than 55 years! Check your Table 2 results. | This has been revised to align better with the result. It now reads, “Those aged more than 55 years were more likely to be hesitant about COVID-19 vaccine uptake. This may be due to limited information about the vaccine or some myth about the vaccine. Being a new disease, the younger individuals who are more on social media are more likely to access more information about the disease and may be more accepting of the vaccine. Other studies have shown that younger age groups were however more hesitant as compared to the elderly (28).” |
| 10 | Line 276: “statistically significant predictor of the hesitancy of the COVID-19 vaccine” kindly replace with association- a cross sectional data can not give you a predictor. You may wish to read more about predictive models. | This has been updated |
| 11 | Line 278: “Urban dwellers were usually more likely to have mobile phones and engage in social media and more likely to obtain information from other sources” This statement is hanging? Not link with the predecessor and how does it lead to VH? | Thanks for the comment, the sentence has been improved on line 288 and 289 |
| 12 | Line 280: “Our study is not without limitations. Our sample size was small and included only two of Liberia’s fifteen counties. Nevertheless, these counties accounted for 83% of all reported COVID-19 cases in the country” These statements are not limitation. Your sample size of 800 plus is adequately powered and sampling two major districts comprising of urban and rural areas out of 15 should provided an adequate representation and can not be a study limitation. Your study limitations may be lack of in-depth analysis of reasons for VH, which will require a qualitative study approach and also lack of follow up to see how many people will eventually get vaccinated. | Thanks for your observation, this section has been revised as suggested. It now reads, “Our study is not without limitations. A qualitative arm would have helped collect in-depth information to help understand other possible reasons for the hesitancy observed. Being only quantitative in nature, our study was not able to explore these. Also, we were not able to follow up with the participants to determine those who eventually took the vaccine.” |
| 13 | Line 282: “The survey provides information needed to strengthen and increase the impact of future planned mass vaccination campaigns” Move to the end of conclusion | The line has been added to the conclusion |
| 14 | Overall while I understood time has caught up with your data, kindly compared your key findings with other African studies e.g Babatunde Oluwatosin Ogunbosi et al. COVID-19 vaccine hesitancy in six geopolitical zones in Nigeria: a cross-sectional survey. Pan African Medical Journal. 2022;42(179). 10.11604/pamj.2022.42.179.34135 | Thanks for providing this resource document, it was compared with my results in the discussion section |

| Reviewer # 4 comments |  |  |
| --- | --- | --- |
| 1. | Regarding your conclusion that vaccine hesitancy should be combated through additional information on television and radio since those are the most common sources of information at 53% - this is probably correct but I was not 100% convinced that it was supported by the data presented. Your denominator was the entire study population and I didn't see a breakdown by those who were accepting of vaccination and those who were not. | Thanks for your comment those that were hesitant of taking the vaccine were 29.1 % (255/877). |
| 2. | Lines 129-130: "...communities were selected based on the number of communities and their distribution in each county." I couldn't really tell from this statement what your selection criteria were. Were you looking for a greater number of communities? What type of distribution did you want, and why? | This has been rephrased for clarity. It now reads, “From each stratum, communities were selected using simple random sampling. The number of communities selected was proportionate number of communities in each stratum in each county. Twenty-five communities were selected in all. Finally, seventeen (seven rural and ten urban) communities were selected in Montserrado; and eight (seven rural and one urban) were selected in Nimba using simple random sampling.” |
| 3. | 133-141: I found the description of Stage 2 a little hard to follow and still don't understand how you used the sampling interval to select the second household. Did you randomly select a direction again? | Thanks for the comment more information has been provided in the stage 2 of the sampling technique for clarity. |
| 4. | General: I saw a previous reviewer recommended removing the description about street interviews. I am not sure I agree with this if street interviews formed part of your data. The questions I would ask myself are whether they formed a meaningful proportion of your data, and if you can describe it in a way that addresses the comment about it sounding haphazard. | The data for the street interview was removed to be analyzed with other data collected during the survey. Thanks for the interest in more of our findings. |
| 5. | 186-187: There was another reviewer who asked about the ethics committee and I also had a question about this. Can you confirm that submitting to the regular ethics committee that you normally would outside a public health emergency was not an option? My concern is that an ethics committee formed of leadership from MOH and NPHIL is not what I would expect in the ERC constitution. For example there may not be diverse backgrounds and no lay person on the committee. | Submitting to the regular ethics committee was not an option at the time of the response. Outside of the response, all research is submitted to the IRB for clearance. The leadership of both institutions consisted of the leadership and members of the Incident Management System which were multi-discipline. |
| 6. | 95: Self-employed is not listed in Table 1, only employed and unemployed. | For the purpose of the analysis were group employment status into employed and unemployed. Self employed was merged with employed. Our interest was to check if having a stable source of livelihood is a factor and not the categories of employment. |
| 7. | 194-196: To say most participants were unmarried (41.4%) seems a little misleading considering married participants were 38.1% and co-habiting 15.6%. | Thank you for your comment The statement has been rephrased. |
| 8. | 210-212: Here it says >55 years is more likely to be hesitant than 45-54 but in the abstract you said the opposite. | This has now been rephrased for consistency |
| 9. | Table 2: Interesting that the most hesitancy was seen in the 15-24 age group, but this is not mentioned in the results. | The information has been added to the prose in the results. This was not emphasized because the association was not statistically significant |
| 10. | Table 2: I think your percentages are reversed for hesitancy yes/no in Montserrado County? | Thanks for the comment the percentages have been recalculated and updated in table 2 |
| 11. | Figure 1: This is where I think it might be helpful to stratify by people who are vaccine hesitant those who are not. Is there a difference between those two groups in sources of information? | The data was collected for those who were hesitant to understand the reason they are hesitant so the suggestion may not be feasible at this time. We agree it would be interesting to see the difference but unable to report that at this time |
| 12. | 254-256: Not sure this is supported by the data? In Figure 1, Facebook as a source of information seems pretty similar between urban and rural. | The information was reported as received. Though the source information might be similar the quality of information received and their understanding of the information could be different. |
| 13. | 278-279: Again, I am not seeing this in Figure 1. In fact, it appears that more rural respondents relied on "other" sources of information | The rural communities have other sources of information like town hall meetings, town crier but majority also have access to radio etc. Unfortunately, it was not separated during the data collection |
