# Supplementary material for: COVID-19 vaccine hesitancy among adults in Liberia, April–May 2021
Source: PLoS One. 2024 Apr 17;19(4):e0297089. doi: 10.1371/journal.pone.0297089 (PMC11023583; doi:10.1371/journal.pone.0297089)
Supplement: Attachment — Submitted filename: Response to reviewers.docx [file pone.0297089.s010.docx]

Liberia Field Epidemiology Training Program
African Field Epidemiology Network (AFENET), Monrovia, Liberia.
lblebo@afenet.net

December 20, 2023

Omar Enzo Santangelo, Ph.D.,
Academic Editor,
PLOS ONE

**RE**: COVID-19 Vaccine Hesitancy among adults in Liberia, April-May 2021 [PONE- D-22-17733]

Dear Dr. Santagelo,

We are very grateful to you and the reviewers for the time and efforts you all put in to review our manuscript and for valuable suggestions to improve the understanding of the work.

We have revised the manuscript to address the points raised during the review process. Our point-by-point responses to all comments and suggestions are tabulated on the next page of this letter.

We hope you find our responses satisfactory and the manuscript now suitable for publication in PLOS ONE, even as we appreciate further suggestions where we failed to address observed concerns.

Sincerely,

Lily M. Sanvee-Blebo

Field Epidemiologist
Liberia Field Epidemiology Training Program, AFENET

[On behalf of all authors]

| Section | Comments | Authors response |
| --- | --- | --- |
| Reviewer # 3 |  |  |
| 1 | It seems that comment #1 was misunderstood. Of the 53% who got their information from television and radio, you don't state the breakdown between people who accept the vaccine and those who are hesitant. If all of the people who are hesitant got their information from other sources, it does not seem like the conclusion to focus more on television and radio would be warranted. Since this is one of your primary conclusions, it would be nice to understand this better. | Thank you for the clarification. We have now conducted further analysis on the source of information which showed that the relationship between vaccine hesitancy status and receiving information through the media (Television/radio) was not found to be statistically significant (p=0.0525). Based on this new finding we have now revised the conclusion of the study to remove attention from media. We appreciate this feedback and do hope that the work is now in an acceptable format. Thank you |
